# Effectiveness of Etanercept Biosimilar Initiating for Etanercept-Naive Patients, Using Ultrasound, Clinical, and Biomarker Assessments in Outcomes of Real-World Therapy (ENPORT-NGSK Study): An Interventional, Multicenter, Open-Label, Single-Arm Clinical Trial

**DOI:** 10.3390/jcm14051775

**Published:** 2025-03-06

**Authors:** Remi Sumiyoshi, Shin-ya Kawashiri, Toshimasa Shimizu, Tomohiro Koga, Rieko Kiya, Shigeki Tashiro, Yurika Kawazoe, Shuntaro Sato, Yukitaka Ueki, Takahisa Suzuki, Tamami Yoshitama, Yoshifumi Tada, Naoki Hosogaya, Hiroshi Yamamoto, Atsushi Kawakami

**Affiliations:** 1 Department of Immunology and Rheumatology, Division of Advanced Preventive Medical Sciences, Nagasaki University Graduate School of Biomedical Sciences, Nagasaki 852-8501, Japan; remis@nagasaki-u.ac.jp (R.S.);; 2 Clinical Research Center, Nagasaki University Hospital, Nagasaki 852-8501, Japan; 3 Center for Collaborative Medical Education and Development, Nagasaki University Institute of Biomedical Sciences, Nagasaki 852-8523, Japan; 4 Rheumatic Disease Center, Sasebo Chuo Hospital, Sasebo 857-1195, Japan; 5 Department of Rheumatology, Japanese Red Cross Nagasaki Genbaku Hospital, Nagasaki 852-8511, Japan; 6 Yoshitama Clinic for Rheumatic Diseases, Kagoshima 899-5117, Japan; 7 Department of Rheumatology, Saga University Hospital, Saga 849-8501, Japan

**Keywords:** rheumatoid arthritis (RA), etanercept, biosimilar, musculoskeletal ultrasound (MSUS), biomarkers

## Abstract

**Background/Objectives**: This study aimed to investigate the effectiveness of etanercept biosimilar 1 under real-world clinical conditions in patients with rheumatoid arthritis (RA), using not only clinical evaluation but also musculoskeletal ultrasound (MSUS). **Methods**: This multicenter, interventional, open-label, single-arm clinical trial conducted a 24-week follow-up. Patients with RA with moderate to high disease activity received weekly subcutaneous injections of etanercept biosimilar 1 at 50 mg/dose for 24 weeks. The effectiveness was evaluated with clinical indices and MSUS. **Results**: Twenty-three patients were evaluated during the study period. The primary endpoint involves a change in the Global OMERACT-EULAR Synovitis Score by MSUS in bilateral second–fifth metacarpophalangeal joints from baseline, demonstrating median (IQR) values of 0 (−4, 1), including 4 (1, 9.8) and 2 (0, 5) at baseline and 24 weeks, respectively. The clinical endpoints exhibited a good treatment response, with 15 (68%) and 18 (86%) patients achieving low disease activity or remission at 12 weeks and 24 weeks, respectively. Additionally, MSUS scores improved at both 12 and 24 weeks compared to baseline. The patients who achieved power doppler remission (total power doppler score = 0) at 24 weeks demonstrated a shorter disease duration and no previous use of biological disease-modifying antirheumatic drugs compared to those with no power doppler remission. **Conclusions**: Etanercept biosimilar 1 exhibited significant improvements not only in clinical indices but also in MSUS assessment, indicating its effectiveness at the structural level.

## 1. Introduction

Rheumatoid arthritis (RA) is an autoimmune disease that induces chronic synovitis in multiple joints, causing progressive destructive arthritis and functional disability [1]. Therefore, achieving and maintaining remission as soon as possible after diagnosis is crucial. Specifically, we follow a treatment strategy named treat-to-target (T2T) [2]. First, conventional synthetic disease-modifying antirheumatic drugs (csDMARDs), such as methotrexate (MTX), are initiated after RA diagnosis, but biological DMARDs (bDMARDs) are the next option if remission is not achieved.

The disadvantage of bDMARDs is their high cost. However, biosimilars have been introduced in recent years, thereby reducing the economic burden. In Japan, etanercept biosimilar 1 (etanercept BS “MA”) received marketing approval in January 2018 for the same indication as an etanercept-reference product (RP) (Enbrel). Etanercept biosimilar 1 is a similar biologic to etanercept–RP, and its equivalence in quality, efficacy, and safety during approval was verified and proven in clinical trials that compared it to etanercept–RP [3]. However, other bDMARDs have been effective by clinical indices and musculoskeletal ultrasound (MSUS) [4,5], whereas etanercept biosimilars have been effective only by clinical indices [6]. Providing evidence of their effectiveness in synovitis on MSUS was considered necessary to help clinical practitioners understand the essential equivalence between etanercept biosimilars and etanercept–RP.

MSUS is non-invasive, objective, inexpensive, and repeatable and is superior to clinical disease activity assessment in evaluating RA activity because of its ability to depict synovial inflammation with high sensitivity [7,8]. Additionally, it is a suitable imaging tool for monitoring treatment. Subclinical synovitis may be present in MSUS even in cases of clinical remission. This subclinical synovitis is a crucial finding that is predictive of joint destruction and relapse [9,10,11]. Therefore, accurately assessing disease activity at the joint level using MSUS is important, not just relying on clinical indices that include subjective factors.

The present study aimed to evaluate the effectiveness of etanercept biosimilar 1 using clinical as well as MSUS measures. Additionally, an exploratory analysis of the association between clinical and MSUS measures of response after treatment was conducted.

## 2. Materials and Methods

### 2.1. Patients

This prospective, open-label, interventional single-arm clinical trial was conducted at Nagasaki University Hospital, Sasebo Chuo Hospital, Japanese Red Cross Nagasaki Genbaku Hospital, Yoshitama Clinic for Rheumatic Diseases, and Saga University Hospital. This study included 23 patients with RA receiving an intervention for 24 weeks.

Inclusion criteria were (1) patients aged ≥20 years upon obtaining informed consent, (2) patients with RA fulfilling the American College of Rheumatology (ACR)/European Alliance of Associations for Rheumatology (EULAR) classification criteria for RA (2010) [12], (3) patients who were assessed by the physician to have moderate to high disease activity upon obtaining consent, (4) patients with Disease Activity Score—28 for Rheumatoid Arthritis with C-reactive protein (DAS28-CRP) of >3.2 at case enrollment, and (5) patients who were fully informed about their participation in this study and gave their written consent.

The exclusion criteria were (1) patients currently receiving oral prednisolone of >7.5 mg/day upon case enrollment, (2) patients with etanercept biosimilar 1 contraindication, (3) patients who had previously used an etanercept–RP, (4) patients who had previously used etanercept biosimilar, (5) patients under treatment with biological agents and JAK inhibitors for RA, except for denosumab, (6) patients whose prednisolone or anti-rheumatic drug usage and dosage were changed within 4 weeks before case enrollment, (7) patients who were treated with banned drugs or banned therapies within 4 weeks before case enrollment, (8) women who were currently pregnant, breastfeeding, or unwilling to follow a medically approved contraceptive plan during the study period, and (9) patients who were judged unsuitable for this study by the investigator.

A patient was terminated prematurely for the following reasons (patients who discontinued before completing the trial were not be replaced): (1) the patient experienced a clinical relapse, (2) etanercept biosimilar 1 needed to be reduced or withdrawn for more than 3 weeks for some reason(s), (3) patients were judged to be unable to continue participation due to adverse event(s), (4) the patient requested to discontinue participation in the study, (5) when the Principal Investigator or a Research Assigning Physician determined that the continuation of the study would be inappropriate for the subject concerned.

The certified review board (CRB) of Nagasaki University approved this study (CRB approval number: CRB7180001), which was registered in the Japan Registry of Clinical Trials (https://jrct.niph.go.jp, accessed 30 January 2025) as jRCTs071200054. We conducted the study under the principles of the Declaration of Helsinki [13] and the Clinical Trials Act (since February 2019), the Act on the Protection of Personal Information and related regulatory notifications, and this clinical study protocol.

### 2.2. Intervention

Patients with RA with moderate to high disease activity were introduced to etanercept biosimilar 1 within 14 days of case enrollment. Etanercept biosimilar 1 was administered as a weekly subcutaneous injection of 50 mg for 24 weeks. All the patients were required to maintain the same csDMARD and oral corticosteroid doses throughout the study period as they were taking before the study. The following treatments were prohibited during the study period: bDMARD or JAK inhibitor administration, concomitant immunosuppressant use (azathioprine, cyclophosphamide, and cyclosporine), or an oral corticosteroid equivalent to >7.5 mg/day of prednisolone, and intra-articular corticosteroid injections.

### 2.3. Outcome Measurements

Study visits took place at baseline and after 12 weeks and 24 weeks of treatment. Supplementary Figure S1 presents the assessments. Clinical physicians were blinded to joint evaluations by MSUS.

Each of the attending physicians assessed clinical disease activity based on the values of the DAS28-CRP, the DAS28-erythrocyte sedimentation rate (ESR) [14], the simplified disease activity index (SDAI) [15], and the clinical disease activity index (CDAI) [16]. The Health Assessment Questionnaire–Disability Index (HAQ-DI) was conducted to evaluate patients’ functional assessment [17].

Participants underwent imaging by MSUS at baseline, 12 weeks, and 24 weeks. A systematic multiplanar grayscale (GS) and power Doppler (PD) examination of each patient’s joints was conducted with a multifrequency linear transducer (12–24 MHz). PD was utilized according to which Doppler modality was the most sensitive on the individual machines. The Doppler settings were adjusted at each hospital following published recommendations [18], and standardized joint and probe positions were used according to guidelines published by the Japan College of Rheumatology (JCR). MSUS evaluation was performed at each participating hospital by JCR-certified sonographers as previously described [19]. Articular synovitis was evaluated with MSUS at dorsal views of 22 joints: bilateral wrist joints, the first–fifth metacarpophalangeal (MCP) joints, the interphalangeal joints, and the second–fifth proximal interphalangeal joints. Each joint was scored for GS as well as PD on a scale of 0–3 semiquantitatively. The sum of the GS or PD scores was considered the total GS or PD scores, respectively. Additionally, we assessed the Outcome Measures in Rheumatology (OMERACT)-EULAR combined PDUS score (i.e., the combined PD score) and the Global OMERACT-EULAR Synovitis Score (GLOESS) [5,20]. The combined PD score was combined with synovial hypertrophy demonstrated by GS and PD [5,20]. Interobserver reliability was confirmed in a previous investigation [21]. PD remission was defined as a PD score = 0 from 22 joints as described previously [19,22].

X-ray imaging of bilateral hands (posteroanterior view) and feet (anteroposterior view) was performed. Joint damage progression was assessed based on the van der Heijde-modified total Sharp score (vdH-mTSS) method by trained JCR-certified rheumatologists (T.K and T.S) as previously described [23], including 16 areas in each hand for erosions and 15 for joint-space narrowing [24].

### 2.4. Biomarker Measurements

The patients’ serum concentrations of the following biomarkers were measured: rheumatoid factor (RF) by a latex agglutination turbidimetric immunoassay (LZ test “Eiken” RF) (Eiken Chemial, Tochigi, Japan); anti-cyclic citrullinated peptide antibodies by a chemiluminescent immunoassay (STACIA MEBLux test CCP) (Medical & Biological Laboratories Co., Ltd., Tokyo, Japan); matrix metalloproteinase-3 (MMP-3) by a latex turbidimetric immunoassay (LTIA) (Panaclear MMP-3 “Latex”) (Sekisui Medical Company Limited, Tokyo, Japan); multiplex cytokine/chemokine bead assays with diluted serum supernatants and a MILLIPLEX MAP Human Cytokine/Chemokine Magnetic Bead Panel (Merck Millipore, Billerica, MA, USA)-Bio-Plex Pro Human Cytokine Assays (Bio-Rad, Hercules, CA, USA) analyzed with a Bio-Plex^®^ MAGPIX™ Multiplex Reader (Bio-Rad), following the manufacturer’s instructions.

The cytokines/chemokines that were measured by the bead panel included interleukin (IL)-1α, IL-1β, IL-1 receptor antagonist, IL-2, IL-3, IL-4, IL-5, IL-6, IL-7, IL-8, IL-9, IL-10, IL-12 (p40), IL-12 (p70), IL-13, IL-15, IL-17, interferon-gamma (IFN-γ), IFN-α2, CXCL1 (growth-related oncogene), granulocyte-macrophage colony-stimulating factor (GM-CSF), granulocyte CSF (G-CSF), CX3CL1 (fractalkine), flt-3 ligand, fibroblast growth factor-2, eotaxin, epidermal growth factor, soluble CD40 ligand, vascular endothelial growth factor, tumor necrosis factor (TNF)-β, TNF-α, transforming growth factor-α, CCL4 (macrophage inflammatory protein [MIP]-1β), CCL3 (MIP-1α), CCL22 (macrophage-derived chemokine [MDC]), CCL7 (monocyte chemotactic protein-3), CCL2 (monocyte chemotactic protein-1), and CXCL10 (IFN-γ-inducible protein [IP]-10). The serum levels of IL-6 and TNFα were measured with specific enzyme-linked immunosorbent assay (ELISA) kits (R&D Systems, Minneapolis, MN, USA).

### 2.5. Study Endpoints

The primary endpoint included changes from baseline to 24 weeks in the GLOESS by MSUS for bilateral second–fifth MCP joints.

The secondary endpoints of this study were the proportion of study participants meeting low disease activity or remission criteria at 12 weeks and 24 weeks. Additionally, changes in various scores from baseline to 12 weeks and 24 weeks, including total GS score, PD score, GLOESS, DAS28-CRP, DAS28-ESR, SDAI, CDAI, vdH-mTSS, HAQ-DI, and biomarkers were measured.

### 2.6. Statistical Analysis Method

This study aimed to exploratorily assess the association between MSUS assessments and clinical measures. We set the sample size at 40 based on the registration period and the number of cooperating research facilities due to the lack of information required for statistically calculating a sample size. All data are presented as medians and interquartile range (IQR) for continuous variables or as frequencies and percentages for discrete variables. The Wilcoxon signed-rank test was conducted to evaluate continuous variables at baseline and each assessment point. Spearman’s correlation coefficients with 95% confidence intervals (CIs) were calculated as a primary analysis for the correlation between changes in clinical measures (DAS28-CRP) and changes in MSUS assessments (GLOESS of bilateral second–fifth MCP joints). A Wilcoxon rank sum test and Fisher’s exact test were conducted for comparisons between the two groups (with/without clinical remission; with/without PD remission) in the exploratory analysis. Additionally, we calculated the standardized response mean (SRM) as the mean change divided by the standard deviation of the change. No confirmatory testing or adjustment for multiplicity was planned because this was an exploratory study. Reported P-values should be interpreted with caution. Statistical analyses were conducted with R version 4.2.2 (R Project for Statistical Computing, Vienna, Austria), JMP Pro version 17.0 software (SAS Institute Inc., Cary, NC, USA), and Graph Pad Prism version 10.2.2 (GraphPad Software, La Jolla, CA, USA).

## 3. Results

### 3.1. Patients’ Characteristics

The target number of patients was 40, but the enrollment period ended after 23 patients had been registered. One patient was excluded due to a lack of data during discontinuation; thus, the full analysis set (FAS) included 22 patients. One patient discontinued at 12 weeks at the patient’s request; hence, 21 completed the study (Supplementary Figure S2).

Table 1 shows the baseline characteristics. The age of the patients was 62 years (46, 73), and 16 (70%) patients were female. The disease duration was 1 year (0, 6.5), and 21 (91%) patients were RF- and anti-CCP-antibody-positive. Eight (35%) patients had been treated with other bDMARDs, including one with infliximab, one with adalimumab, two with tocilizumab, and six with abatacept (with possible duplicates among the same individuals). Concomitant medications for RA included MTX in 17 (74%) patients at 10 mg/week (8, 12). Concomitant prednisolone was administered in nine (39%) patients at 5 mg/day (2.5, 5).

### 3.2. Primary Endpoint

The primary endpoint (FAS; n = 22), the change in the GLOESS by MSUS in bilateral second–fifth MCP joints from baseline, demonstrated median (IQR) values of 0 (−4, 1), with 4 (1, 9.8) at baseline and 2 (0, 5) at 24 weeks. Additionally, we calculated Spearman’s correlation coefficient [95% CI] for changes in GLOESS of bilateral second–fifth MCP joints and changes in DAS28-CRP, from baseline to 24 weeks: ρ = −0.075 [−0.491, 0.368].

### 3.3. Secondary Endpoints

The secondary outcomes (FAS; n = 22) were as follows. At 12 weeks, 15 (68%) patients achieved low disease activity or remission by DAS28-CRP criteria, with 10 (45%) in remission, which increased to 86% and 67% at 24 weeks, respectively. DAS28-CRP, DAS28-ESR, SDAI, CDAI, and HAQ-DI were assessed at baseline, 12 weeks, and 24 weeks for clinical measures. Additionally, SRM was calculated (Table 2). Median (IQR) results at baseline, 12 weeks, and 24 weeks were 4.3 (4.0, 4.6), 2.6 (2.0, 3.3), and 2.1 (1.9, 3.1) for DAS28-CRP; 5.1 (4.2, 5.6), 2.7 (2.0, 3.4), and 2.5 (1.4, 3.3) for DAS28-ESR; 22 (19, 24), 7.6 (5.1, 12), and 6.6 (3.4, 8.3) for SDAI; 21 (18, 23), 7.2 (4.9, 12), and 6.5 (2.4, 7.6) for CDAI; 0.88 (0.25, 1.5), 0.25 (0, 1.2), and 0.25 (0, 1.2) for HAQ-DI, respectively.

**Figure 1:**
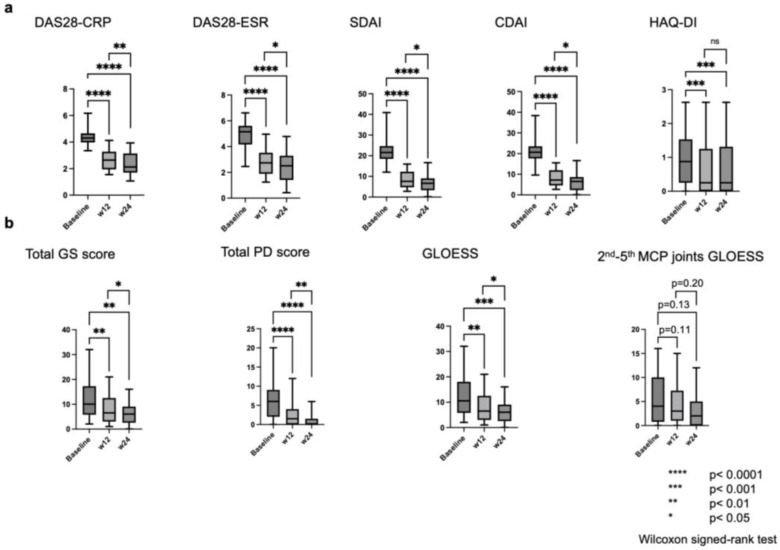
Assessment of effectiveness. (**a**) Assessment of effectiveness in clinical measures. The *p*-values and 95% CI for baseline to 12 weeks, baseline to 24 weeks, and 12–24 weeks were as follows. DAS28-CRP: *p* < 0.0001 [−2.13, −1.35], *p* < 0.0001 [−2.40, −1.53], and *p* < 0.01 [−0.50, −0.02]. DAS28-ESR: *p* < 0.0001 [−2.51, −1.62], *p* < 0.0001 [−2.82, −1.76], and *p* = 0.03 [−0.52, 0.04]. SDAI: *p* < 0.0001 [−17.4, −10.8], *p* < 0.0001 [−18.4, −11.7], and *p* = 0.02 [−2.88, −0.03]. CDAI: *p* < 0.0001 [−16.4, −9.84], *p* < 0.0001 [−17.6, −11.1], and *p* = 0.02 [−3.02, −0.16]. HAQ-DI: *p* < 0.001 [−0.48, −0.13], *p* < 0.001 [−0.45, −0.12], and *p* = 0.93 [−0.11, 0.15]. (**b**) Assessment of effectiveness in MSUS. The *p*-values and 95% CI for baseline to 12 weeks, baseline to 24 weeks, and 12–24 weeks were as follows. Total GS score: *p* < 0.01 [−5.60, −1.13], *p* < 0.01 [−7.58, −1.85], and *p* = 0.03 [−3.34, −0.09]. Total PD Score: *p* < 0.0001 [−6.18, −2.36], *p* < 0.0001 [−7.17, −2.83], and *p* < 0.01 [−1.51, −0.30]. GLOESS: *p* < 0.01 [−5.77, −1.22], *p* < 0.001 [−7.76, −1.96], and *p* = 0.03 [−3.34, −0.09]. GLOESS of bilateral second–fifth MCP joints: *p* = 0.11 [−2.37, 0.10], *p* = 0.13 [−3.38, −0.05], and *p* = 0.20 [−1.76, 0.34]. CDAI, clinical disease activity index; CRP, C-reactive protein; DAS28, Disease Activity Score—28; ESR, erythrocyte sedimentation rate; GLOESS, Global OMERAC EULAR Synovitis Score; GS, gray scale; HAQ-DI, Health Assessment Questionnaire–Disability Index; MCP, metacarpophalangeal; ns, not significant; PD, power Doppler; SDAI, simplified disease activity index.

All clinical measures were improved at 12 weeks and 24 weeks of treatment compared to baseline (Figure 1a). Similar analyses were conducted for the MSUS assessments (Table 2, Figure 1b). The median (IQR) values at baseline, 12 weeks, and 24 weeks for MSUS assessments were 10 (6, 17), 6.5 (3, 12), and 6 (3, 8) for total GS score; 6 (2.2, 9), 1.5 (0, 3.8), and 0 (0, 1) for total PD score; 10 (6, 18), 6.5 (3, 12), and 6 (3, 8) for GLOESS; 4 (1, 9.8), 3 (1, 6.2), and 2 (0, 5) for GLOESS of bilateral second–fifth MCP joints, respectively. Total GS score, total PD score, and GLOESS improved at 12 weeks and 24 weeks of treatment compared to baseline. The median (IQR) comparison of vdH-mTSS was 10 (4.2, 25) at baseline and 11 (6, 26) at 24 weeks. TNF-α was increased after treatment for cytokines and chemokines. The levels were 16 (14, 21) and 63 (56, 94) in the multiplex cytokine array and 11 (9.9, 12) and 140 (96, 180) in ELISA at baseline and 24 weeks, respectively. Supplementary Figure S3 shows the results of the multiple cytokine array.

### 3.4. Exploratory Endpoints

The patients were categorized based on clinical remission status at 24 weeks into two groups, and a univariate analysis was conducted to compare the baseline characteristics of each group (Table 3). The analysis was conducted in 21 cases, excluding one case with a lack of assessment at 24 weeks. The patients who achieved clinical remission at 24 weeks (14 cases) demonstrated a shorter disease duration than those who did not achieve clinical remission (7 cases) (0.5 years [0.0, 4.0] vs. 6.0 years [0.0, 11.0)], respectively, *p* = 0.19) and no previous use of bDMARDs (3/14 [21%] cases vs. 4/7 [57%] cases, respectively, *p* = 0.16), although with no significant differences in baseline patient characteristics. Patients who achieved clinical remission at 24 weeks exhibited lower baseline vdH-mTSS scores (7.8 [1.8, 18.4] vs. 26 [9, 86.5], *p* = 0.048).

Subsequently, the patients were categorized based on whether they achieved PD remission (total PD score = 0) at 24 weeks into two groups, and the baseline characteristics of each group were compared (Table 3). Patients who achieved PD remission at 24 weeks demonstrated a shorter disease duration (0.0 years [0.0, 4.5] vs. 5.0 years [1.5, 14.3], respectively, *p* = 0.0497), and no previous use of bDMARDs (2/13 [15%] cases vs. 5/8 [63%] cases, respectively, *p* = 0.056). The comparison between patients with and without PD remission revealed no differences in baseline measures, but the DAS28-CRP, DAS28-ESR, total PD score, and vdH-mTSS scores were slightly lower in the group that achieved PD remission.

### 3.5. Safety

The safety analysis set (n = 23) consisted of 16 adverse events (AEs) from the start of the study to 24 weeks. Of these, no serious AEs were reported, and the severity of all the AEs was grade 2: moderate (requiring minimal/local/non-invasive treatment for the adverse event). A total of eight cases exhibited adverse drug reactions from the start of the study to 24 weeks. Two (8.7%) herpes zoster cases and one (4.3%) case each of nasopharyngitis, leukocytopenia, liver dysfunction, cellulitis, injection site reaction, and oral herpes was reported. No serious adverse drug reactions were observed. One patient with cellulitis required etanercept biosimilar 1 withdrawal for more than two weeks and the study drug was discontinued. In the one case that exhibited an injection site reaction, the severity was mild, and discontinuation of the etanercept biosimilar 1 was not required, allowing treatment to continue. Additionally, no cases of diminished efficacy leading to drug discontinuation were observed during the study, and no immunogenicity issues were reported throughout the study period.

## 4. Discussion

This study revealed that treatment with etanercept biosimilar 1 in patients with active RA resulted in clinical assessments, demonstrating improvement at 12 weeks in DAS28-CRP, DAS28-ESR, SDAI, and CDAI scores, with additional improvements observed at 24 weeks. The total GS score, total PD score, and GLOESS exhibited improvement at 12 and 24 weeks compared to baseline in the MSUS measures.

Etanercept biosimilars have demonstrated comparable clinical efficacy to their RP in phase III clinical trials [3], but the products cannot be completely identical due to the complex biological manufacturing process [25]. Etanercept biosimilars have generally been used for some time and comparisons have been conducted between etanercept RP and biosimilars in real-world clinical practice. A Romanian study that involved 123 patients on etanercept–RP and 119 patients on etanercept biosimilars revealed no difference in DAS28 remission or EULAR response in a composite analysis of both biologic-naïve and biologic-experienced patients [26]. A cohort study conducted in Portugal indicated that the treatment continuation rate, the proportion of patients who achieved remission, and those who attained good EULAR responses were comparable between etanercept–RP and its biosimilar [27]. Additionally, a United Kingdom study compared outcomes after 6 and 12 months of treatment among patients with RA who started either etanercept–RP or etanercept biosimilar as their first biologic and revealed that the proportion of patients achieving DAS28 remission and EULAR response rates was similar between the treatments [28]. These results indicate that etanercept–RP and biosimilars are equally effective in real-world clinical practice. However, no prospective studies assessing the effectiveness of etanercept biosimilar, including MSUS, have yet been reported. The strength of this study lies in its prospective assessment of therapeutic effectiveness using both clinical assessments and MSUS to precisely and objectively assess disease activity at the joint level in patients from multiple centers with a standardized uniform MSUS evaluation. The APPRAISE study [5], which assessed the efficacy of abatacept in patients with RA who had an inadequate response to MTX, revealed similar results in the GLOESS of 22 joints and GLOESS of both second–fifth MCP joints in MSUS assessment. This indicated the rapid action of abatacept regardless of the number of joints evaluated. Therefore, this study followed the APPRAISE study design and set GLOESS of second–fifth MCP joints as the primary endpoint. Our study revealed no improvement in the GLOESS of second–fifth MCP joints, but the GLOESS of 22 joints indicated enhancement, which could be attributed to the small number of evaluated joints and the small number of cases. We performed a correlation between changes in clinical and MSUS assessments. We calculated Spearman’s correlation coefficient [95% CI] for changes in GLOESS of bilateral second–fifth MCP joints and changes in DAS28-CRP as a primary analysis. Additionally, we analyzed the correlation between changes in the DAS28-CRP and GLOESS of 22 joints, DAS28-CRP and total PD score, and DAS28-CRP and total GS scores. Similar analyses were conducted for DAS28-ESR, SDAI, and CDAI, but with unclear correlations. This may have been because SRM revealed a greater improvement in scores in the clinical assessments than in MSUS evaluations. This indicates the existence of cases with a discrepancy between the results of clinical and MSUS assessments. Cases with clinical remission but residual activity in MSUS, as well as the opposite, were considered to have occurred. Particular attention should be paid to cases with clinical remission but residual activity on MSUS, as this may indicate a potential for future joint destruction [9,10,11].

The cytokine/chemokine that showed a significant change before and after treatment was TNF-α. Previous studies have reported that detectable levels of TNF-α in the blood increase after TNF-α inhibitor administration, primarily due to the formation of TNF-α complexes with the TNF-α inhibitor [29,30]. We consider that a similar phenomenon occurred in the present study.

Patients who achieved PD remission (total PD score = 0) at 24 weeks demonstrated shorter disease durations and a higher proportion of patients receiving their first biologics. Baseline disease activity exhibited no difference but a tendency to be lower. No significant differences were found in baseline RF, MMP-3, or anti-CCP antibodies (*p* = 0.91, *p* = 0.59, *p* = 0.64, respectively). The early introduction of etanercept biosimilar 1 may improve PD signals in MSUS early on and inhibit the progression of joint destruction. The results provide useful evidence to support the clinical use of etanercept biosimilar 1 at the same level as etanercept–RP.

This study had several limitations. First, the sample size was small and there was no control group. Although this was a prospective study registered across multiple centers, the statistical power of the results was limited, making it difficult to draw definitive conclusions about the effectiveness of the treatment in a broader patient population. Secondly, the treatment was introduced in a real-world situation, and cases with a relatively short disease duration were included, which may have caused a good treatment response. Additionally, the present study only assessed patients for up to 24 weeks. In the future, long-term follow-up studies will be necessary to assess the persistence of remission and structural benefits, as well as economic analyses to evaluate the cost-effectiveness of biosimilar adoptions in routine practice.

## 5. Conclusions

The effectiveness of etanercept biosimilar 1 was assessed in real-world clinical practice for patients with RA with moderate to high disease activity. It exhibited improvement not only in clinical assessment but also in activity measured by MSUS. This is the first prospective study to utilize MSUS for assessing the effectiveness of etanercept biosimilar 1.

## Figures and Tables

**Table 1:** Baseline characteristics.

	n = 23
Age, years	62 (46, 73)
Sex, female	16 (70)
Height, cm	154 (149, 161)
Weight, kg	55 (52, 56)
Disease duration, year	1.0 (0.0, 6.5)
Rheumatoid-factor-positive	21 (91)
Anti-CCP-antibody-positive	21 (91)
Smoking history *	12 (52)
Current smoker	6 (50)
Former smoker	6 (50)
Inhaled cigarettes, day	15.0 (9.8, 20.0)
Duration of smoking, years **	34 (22, 40)
Complications of hypertension	7 (30)
Complications of osteoporosis	6 (26)
Complications of osteoarthritis	4 (17)
Previous use of bDMARDs	13 (57)
Infliximab †	1 (4.3)
Adalimumab †	1 (4.3)
Tocilizumab †	2 (8.7)
Abatacept †	6 (26)
Concomitant medications	
Methotrexate	17 (74)
Methotrexate dose, mg/week	10 (8.0, 12.0)
Prednisolone	9 (39)
Prednisolone dose, mg/day	5 (2.5, 5.0)

Data are shown as n (%) or median (Interquartile range); bDMARDs, biological disease-modifying anti-rheumatic drugs; CCP, cyclic citrullinated peptide; † denominator of percentage is 23 patients; * missing: n = 11, ** missing: n = 17.

**Table 2:** Evaluation of effectiveness.

Visit	Median	IQR	SRM
DAS28-CRP			
Changes 0–12 weeks	−1.6	(−2.4, −1)	−2
Changes 0–24 weeks	−1.8	(−2.6, −1.2)	−2.1
DAS28-ESR			
Changes 0–12 weeks	−2.2	(−2.8, −1.2)	−2
Changes 0–24 weeks	−2.2	(−3.1, −1.2)	−2
SDAI			
Changes 0–12 weeks	−12	(−18, −8.9)	−1.9
Changes 0–24 weeks	−13	(−19, −11)	−2.1
CDAI			
Changes 0–12 weeks	−12	(−18, −7.8)	−1.8
Changes 0–24 weeks	−14	(−19, −10)	−2
HAQ-DI			
Changes 0–12 weeks	−0.19	(−0.5, 0)	−0.77
Changes 0–24 weeks	−0.12	(−0.5, 0)	−0.77
Total GS score			
Changes 0–12 weeks	−1.5	(−5.8, −0.25)	−0.67
Changes 0–24 weeks	−2	(−6, −1)	−0.75
Total PD score			
Changes 0–12 weeks	−3.5	(−6, −1.2)	−0.99
Changes 0–24 weeks	−4	(−8, −2)	−1
GLOESS			
Changes 0–12 weeks	−1.5	(−5.8, −0.25)	−0.68
Changes 0–24 weeks	−3	(−7, −1)	−0.76
2nd–5th MCP joints GLOESS			
Changes 0–12 weeks	−0.5	(−1, 0)	−0.41
Changes 0–24 weeks	0	(−4, 1)	−0.47
vdH-mTSS			
Changes 0–24 weeks	0	(0, 0.5)	

CDAI, clinical disease activity index; CRP, C-reactive protein; DAS28, Disease Activity Score—28; ESR, erythrocyte sedimentation rate; GLOESS, Global OMERACT-EULAR Synovitis Score; GS, gray scale; HAQ-DI, Health Assessment Questionnaire–Disability Index; IQR, interquartile range; MCP, metacarpophalangeal; vdH-mTSS, van der Heijde-modified total Sharp score; PD, power Doppler; SDAI, simplified disease activity index; SRM, standardized response mean.

**Table 3:** Comparisons of baseline characteristics between remission and non-remission patients.

**Baseline** **Characteristics**	**No Clinical Remission Achieved at 24 Weeks,** **n = 7**	**Clinical Remission Achieved at 24 Weeks,** **n = 14**	***p*-Value**
Age, years	61 (49, 76)	64 (44, 74)	1
Female sex	6 (86)	8 (57)	0.97
Height, cm	152 (147, 160)	154 (151, 162)	0.31
Weight, kg	57 (56, 66)	54 (51, 56)	0.09
Disease duration, year	6 (0, 11)	0.5 (0, 4)	0.19
Rheumatoid-factor-positive	6 (86)	13 (93)	1
Anti-CCP-antibody-positive	6 (86)	13 (93)	1
Previous use of bDMARDs	4 (57)	3 (21)	0.16
Total GS score	10 (6, 17)	10 (4, 17)	0.85
Total PD score	6 (3, 9)	5 (1.8, 9.8)	0.74
GLOESS	11 (6, 18)	10 (4, 17.8)	0.71
DAS28-CRP	4.3 (4.1, 4.6)	4.2 (3.6, 4.7)	0.74
DAS28-ESR	5.3 (4.3, 5.6)	5.1 (3.9, 5.6)	0.63
SDAI	22 (20, 25)	21 (17, 23)	0.46
CDAI	22 (18, 25)	20 (17, 22)	0.71
HAQ-DI	1.38 (0.25, 1.63)	0.88 (0.10, 1.38)	0.48
vdH-mTSS	26 (9, 86.5)	7.8 (1.8, 18.4)	0.048
**Baseline** **Characteristics**	**No PD Remission Achieved at 24 Weeks,** **n = 8**	**PD Remission Achieved at 24 Weeks,** **n = 13**	***p*-Value**
Age, years	60 (50, 81)	65 (38, 72)	0.66
Female sex	5 (63)	9 (69)	0.79
Height, cm	152 (148, 162)	154 (149, 161)	0.74
Weight, kg	56 (54, 58)	54 (51, 61)	0.56
Disease duration, year	5.0 (1.5, 14.3)	0.0 (0.0, 4.5)	0.0497
Rheumatoid-factor-positive	6 (75)	13 (100)	0.13
Anti-CCP-antibody-positive	6 (75)	13 (100)	0.13
Previous use of bDMARDs	5 (63)	2 (15)	0.056
Total GS score	10.5 (7, 17.8)	10 (4.5, 15.5)	0.42
Total PD score	7.5 (3.8, 9)	4 (2, 8.5)	0.36
GLOESS	11 (7, 18)	10 (4.5, 16)	0.36
DAS28-CRP	4.4 (4.1, 5.2)	4.2 (3.6, 4.6)	0.26
DAS28-ESR	5.4 (4.5, 5.9)	5.0 (3.6, 5.4)	0.1
SDAI	21 (19, 30)	22 (18, 24)	0.8
CDAI	19 (17, 30)	20 (18, 22)	0.94
HAQ-DI	1.25 (0.25, 1.84)	0.75 (0.13, 1.56)	0.54
vdH-mTSS	21 (4.6, 73.9)	9 (3.5, 19.3)	0.38

Data are shown as n (%) or median (Interquartile range); CCP, cyclic citrullinated peptide; CDAI, clinical disease activity index; CRP, C-reactive protein; DAS28, Disease Activity Score—28; ESR, erythrocyte sedimentation rate; GLOESS, Global OMERACT-EULAR Synovitis Score; GS, gray scale; HAQ-DI, Health Assessment Questionnaire–Disability Index; vdH-mTSS, van der Heijde-modified total Sharp score.

## Data Availability

The datasets used or analyzed (or both) during the current study are available from the corresponding author upon reasonable request.

## Supplementary Materials

The following supporting information can be downloaded at: https://www.mdpi.com/article/10.3390/jcm14051775/s1, Supplementary Figure S1. Study schedule for the outcome measurements. ACPA, anti-cyclic citrullinated peptide antibody; CRP, C-reactive protein; DAS28, Disease Activity Score—28; ESR, erythrocyte sedimentation rate; HAQ-DI, Health Assessment Questionnaire–Disability Index; MMP-3, matrix metalloproteinase-3; RF, rheumatoid factor; VAS: Visual Analog Scale. Supplementary Figure S2. Patient flow chart. Supplementary Figure S3. The results of the multiple cytokine and chemokine array.

## Abbreviations

The following abbreviations are used in this manuscript:

ACPA	Anti-cyclic citrullinated peptide antibodies
ACR	American College of Rheumatology
AEs	Adverse events
bDMARDs	Biological disease-modifying anti-rheumatic drugs
CDAI	Clinical disease activity index
CI	Confidence interval
CRB	Certified review board
CRP	C-reactive protein
csDMARDs	Conventional synthetic disease-modifying antirheumatic drugs
DAS28	Disease Activity Score—28
ELISA	Enzyme-Linked Immuno Sorbent Assay
ESR	Erythrocyte sedimentation rate
EULAR	European League Against Rheumatism
FAS	Full analysis set
GLOESS	Global OMERACT-EULAR Synovitis Score
GS	Gray scale
HAQ-DI	Health Assessment Questionnaire–Disability Index
IL	Interleukin
IP	Interphalangeal
IQR	Interquartile range
JAK	Janus kinase
JCR	Japan College of Rheumatology
MCP	Metacarpophalangeal
MMP-3	Matrix metalloproteinase-3
MSUS	Musculoskeletal ultrasound
MTX	Methotrexate
OMERACT	Outcome Measures in Rheumatology
PD	Power Doppler
PIP	Proximal interphalangeal
PSL	Prednisolone
RA	Rheumatoid arthritis
RF	Rheumatoid factor
RP	Reference product
SDAI	Simplified disease activity index
SRM	Standardized response mean
TNF	Tumor necrosis factor
T2T	treat-to-targe
vdH-mTSS	Van der Heijde-modified total Sharp score
