# Supplementary material for: Effectiveness of Etanercept Biosimilar Initiating for Etanercept-Naive Patients, Using Ultrasound, Clinical, and Biomarker Assessments in Outcomes of Real-World Therapy (ENPORT-NGSK Study): An Interventional, Multicenter, Open-Label, Single-Arm Clinical Trial
Source: J Clin Med. 2025 Mar 6;14(5):1775. doi: 10.3390/jcm14051775 (PMC11900585; doi:10.3390/jcm14051775)
Supplement: Supplementary file 1 [file jcm-14-01775-s001.zip › jcm-3479023-supplementary.pdf]

Study phase	Baseline Screening	Treatment 50mg once weekly			Discontinuation
Study week	Weeks −2 to 0	Week 0	Week 12	Week 24	Weeks 0-24
Inclusion/exclusion	●	-	-	-	-
Demographics	●	-	-	-	-
Enrollment	●	-	-	-	-
Research drug administration	-	←	—	→	-
Concomitant medications/ combination therapy	←	—	—	—	→
Adverse events	-	←	—	—	→
Vital signs	●	●	●	●	●
DAS28 joint assessment	●	●	●	●	●
VAS assessments：Patient global	●	●	●	●	●
VAS assessment: Physician global	●	●	●	●	●
DAS28-ESR, DAS28-CRP	●	●	●	●	●
HAQ-DI	●	●	●	●	●
Laboratory: Blood chemistry	●	●	●	●	●
Laboratory: Hematology	●	●	●	●	●
Laboratory: RF・ACPA・MMP-3	-	●	●	●	●
Laboratory: Cytokines	-	●	-	●	●
Musculoskeletal ultrasound	-	●	●	●	●
X-ray	-	●	●	●	-

ACPA: anti-cyclic citrullinated peptide antibody, CRP: C-reactive protein, DAS28: Disease Activity Score 28, ESR: erythrocyte sedimentation rate, HAQ-DI: Health Assessment Questionnaire Disability Index, MMP-3: matrix metalloproteinase-3, RF: rheumatoid factor, VAS: Visual Analog Scale.

Supplementary Figure S1. Study schedule for the outcome measurements. ACPA, anti-cyclic citrullinated peptide antibody; CRP, C-reactive protein; DAS28, Disease Activity Score—28; ESR, erythrocyte sedimentation rate; HAQ-DI, Health Assessment Questionnaire–Disability Index; MMP-3, matrix metalloproteinase-3; RF, rheumatoid factor; VAS: Visual Analog Scale.

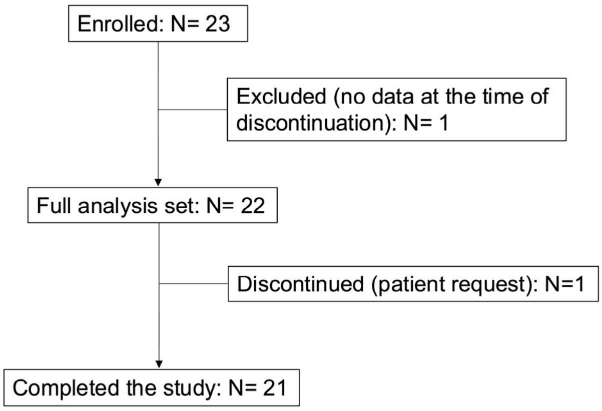

Supplementary Figure S2. Patient flow chart.

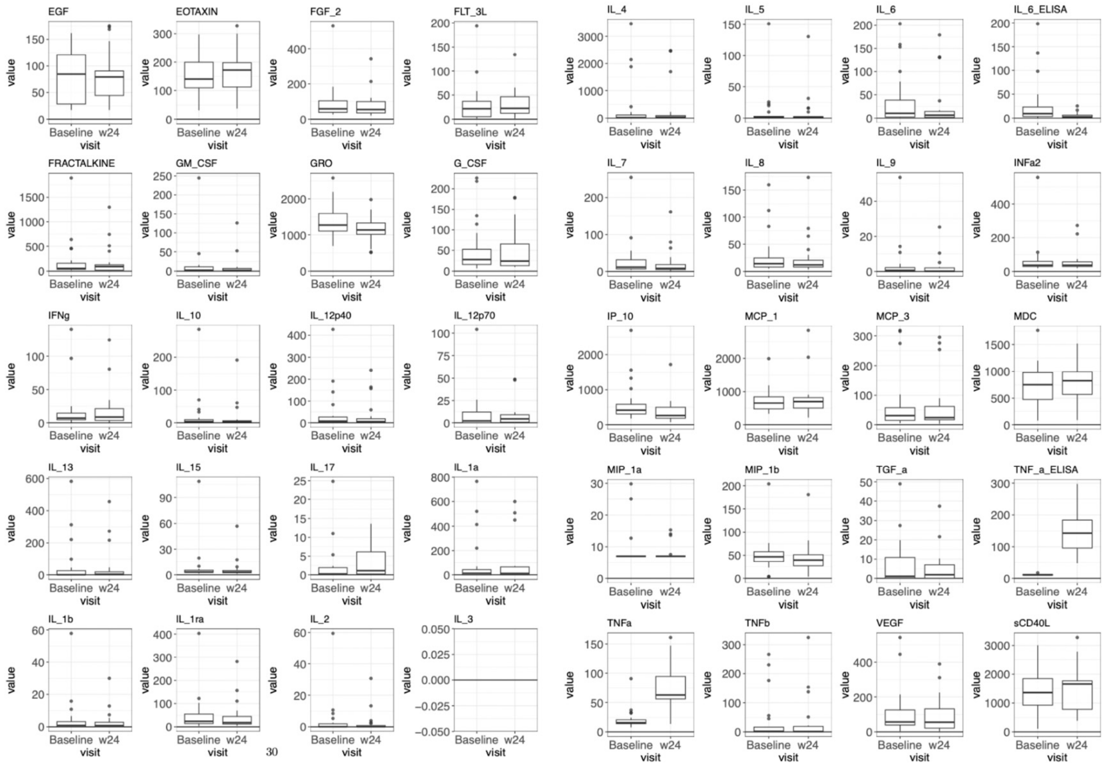

Supplementary Figure S3. The results of the multiple cytokine and chemokine array.
